# Internal Perspectives on Visual Identities in Higher Education: A Case Study of Top-Ranked Universities in Indonesia

**DOI:** 10.12688/f1000research.159232.2

**Published:** 2025-03-26

**Authors:** Putri Dwitasari, Ellya Zulaikha, Syarifa Hanoum, Rabendra Yudistira Alamin, Luqman Lee

**Affiliations:** 1 Interdisciplinary School of Management & Technology, Institut Teknologi Sepuluh Nopember, Surabaya, East Java Province, Indonesia; 2 Visual Communication Design, Faculty of Creative Design and Digital Business, Institut Teknologi Sepuluh Nopember, Surabaya, East Java Province, Indonesia; 3 The Design School, Faculty of Innovation and Technology, Taylor's University, Subang Jaya, Selangor, Malaysia

**Keywords:** Visual identity, reputation, internal stakeholder perception, experiential brand meaning, HEIs

## Abstract

**Background:**

The neglect of visual identity (VI) at the organizational level within higher education institutions (HEIs) has become a critical issue, while previous studies over the past decade has focused on HEI branding and reputation. This creates a potential gap in understanding HEI branding processes. Thus, this study aims to explore the relationship between VI and HEI reputation by integrating the Expressiveness Quotient (EQ) and experiential brand meaning at the organizational level.

**Method:**

Using a qualitative case study approach, the study involves semi-structured interviews with 29 employees from five top-ranked universities in Indonesia. Furthermore, it analyzes the integration of experiential brand meaning across the stages of awareness, interpretation, appropriation, and communication, alongside the EQ framework to assess how visual identity impacts visibility, distinctiveness, transparency, authenticity, and consistency.

**Result:**

The findings indicate that visual identity significantly influences perceptions of institutional identity and reputation. Effective management of visual identity elements enhances competitive advantage in academia and aligns internal stakeholder perceptions with external branding, which is essential for a cohesive organizational identity.

**Conclusion:**

This study emphasizes the strategic importance of visual identity in enhancing institutional reputation and provides a model for universities aiming to strengthen their reputational power through effective visual identity management. The study also reveals strong awareness and acceptance of brand identity.

## 1. Introduction

Higher Education Institutions (HEIs) have increasingly focused on reputation and brand image to strengthen their competitiveness in university rankings, both nationally and internationally. This focus aids in differentiating HEIs from competitors to meet the need for articulating institutional essence within an increasingly competitive market ( Balaji et al., 2016). Furthermore, reputation and brand image are acknowledged by HEIs as essential elements that can create distinct characteristics within higher education, supporting the commercialization of products (i.e., academic programs) and services ( Clark et al., 2020; Mohamad et al., 2017). Additionally, reputation and brand image in higher education have been identified as critical areas in the context of future theory and practice ( Clark et al., 2020; Melewar & Nguyen, 2014). Although HEIs exert efforts to build a reputation and brand image recognized for enhancing competitiveness, these efforts also require organizational activities to ensure that brand values projected to the market are upheld and conveyed by employees. Dean et al. (2016) explored how personnel collectively create brand meaning through their experiences and social interactions within the university context. In this case, the specific focus on internal branding, through strengthening identity, remains largely overlooked ( Clark et al., 2020).

The foundational constructs of HEI branding, such as identity and image, and their strong interaction with reputation, remain underexplored at the organizational level ( Gregersen & Johansen, 2022). In this context, Visual Identity (VI) plays a critical role in shaping a university’s reputation, serving as a tangible representation of the institution’s values, mission, and quality ( Baker & Balmer, 1997; Hunter, 2020; Melewar et al., 2005; Mula-Falcón et al., 2024). Gregersen and Johansen (2022) highlighted in their study the oversight by HEIs in understanding reputation and branding, often neglecting VI, which may result in short-term performance. However, VI’s role in organizational communication is increasingly recognized, especially with the emergence of the “visual shift” in communication research, which emphasizes the importance of images and visual elements in capturing public attention ( Verčič & Wisenberg, 2021; Crăciun, 2019). In conveying the unique character and values developed by HEIs, VI is directed toward various stakeholders, including media, academics, corporations, government, and alumni, while internal stakeholders include students, faculty, and staff ( Dean et al., 2016; Melewar et al., 2005). Implicitly, Khan and Hemsley-Brown (2024) have investigated the impact of VI on course design values, a key factor influencing students’ expectations. In this context, VI and HEI reputation play essential roles in shaping students’ perceptions of the values offered by the institution. Therefore, through its graphical and symbolic elements, VI helps communicate the essence and values of higher education institutions to students, who, in turn, form perceptions of HEI quality ( Coman et al., 2021; Dean et al., 2016; Spry et al., 2020).

In this vein, managing effective communication with both external and internal stakeholders is crucial to strengthening the brand image of HEIs. Brand identity reflects internal perception, while brand image reflects external viewpoints, highlighting the need for consistent internal branding to support external brand communication ( Aaker, 2012; Clark et al., 2020). Meanwhile, HEIs emphasize the importance of aligning brand personality with organizational culture, particularly through internal communication that involves employees in maintaining unified branding efforts. Slade-Brooking (2016) highlights that a robust internal communication strategy not only articulates but also reinforces brand promises. Employees, as key internal stakeholders, shape public perception by fostering trust through an integrated understanding of brand identity. Research by Bolhuis et al. (2018) supports this, showing that employees’ identification with the organization strengthens when the visual identity aligns authentically with institutional values and culture, as also supported by other studies ( Miotto et al., 2020; Wu and Cheong, 2024). This alignment, as noted by Frandsen and Huzzard (2021), helps to avoid potential dissonance within the academic environment that could hinder external brand formation.

A broader comparison with global standards would significantly enhance this study by providing a more comprehensive perspective on VI and branding practices in HEIs. While Indonesian universities emphasize reputation-building and brand image strategies, HEIs in other regions have adopted more structured approaches that integrate VI into their strategic branding frameworks ( Melewar & Nguyen, 2014). For example, European universities, particularly in the UK and Scandinavia, have embedded VI within their institutional governance, ensuring consistency in visual representation across various communication channels ( Foroudi et al., 2019; Gregersen & Johansen, 2022; Lafuente-Ruiz-de-Sabando et al., 2018). Similarly, leading American HEIs incorporate VI within digital branding initiatives, leveraging social media and multimedia platforms to enhance stakeholder engagement and institutional recognition ( Hemsley-Brown & Oplatka, 2022; Wu & Cheong, 2024). Furthermore, in Asian universities emphasize VI as part of a broader cultural and academic identity, using visual elements to reinforce national heritage while appealing to global audiences ( Manzoor et al., 2021; Shahjahan et al., 2022). Incorporating these global benchmarks could provide valuable insights for Indonesian universities, particularly in aligning their VI strategies with best practices in international higher education branding ( Clark et al., 2020; Mohamad et al., 2017). Additionally, a comparative analysis would highlight gaps and opportunities in the current approach, offering a more nuanced understanding of how Indonesian HEIs can enhance their competitiveness on a global scale.

Additionally, various factors such as organizational culture, developmental stage, resources, political dynamics, and student demographics further influence HEIs’ branding strategies ( Asaad et al., 2013). HEIs in Indonesia, for instance, as part of a developing country, continue to create a dynamic institutional environment, where regulations play a role in shaping reputation management approaches. For example, specific regulations designate certain universities as “Legal Entity Universities” (Perguruan Tinggi Berbadan Hukum, or PTN-BH), focusing on maintaining and enhancing institutional reputation. There are 21 universities operating as PTN-BH in Indonesia, but only five PTN-BH universities rank highest in the Quacquarelli Symonds World University Rankings (QS WUR), Times Higher Education (THE), and Webometrics: Universitas Indonesia (UI), Universitas Gadjah Mada (UGM), Institut Teknologi Bandung (ITB), Universitas Airlangga (Unair), and Institut Teknologi Sepuluh Nopember (ITS). Dennis et al. (2016) explain that university rankings—including QS World University Rankings, Times Higher Education, academic rankings, and national rankings—are key metrics reflecting institutional reputation. These rankings have become a focal point for assessing quality and reputation, as noted by Dwitasari et al. (2022), emphasizing the competitive context in which universities strive to position themselves more favorably.

Thus, this study highlights the significance of HEI reputation and identifies a considerable gap specifically in addressing brand development and management related to visual identity and reputation, also underscored by Clark et al. (2020). Meanwhile, in existing research, there remains a lack of understanding of HEI branding flows, which primarily prioritize brand image and reputation (e.g., Del-Castillo-Feito et al., 2019; Lafuente-Ruiz-de-Sabando et al., 2018; Miotto et al., 2020), with VI often overlooked at the organizational level ( Gregersen & Johansen, 2022). Therefore, this study aims to explore the level of knowledge, understanding, and employee engagement in recognizing the institution’s brand identity across five PTN-BH universities. The theoretical framework used is the Expressiveness Quotient (EQ) developed by Fombrun and Van Riel (2004), which evaluates institutional reputation through five dimensions: visibility, distinctiveness, transparency, authenticity, and consistency. These dimensions help organizations gauge emotional appeal and stakeholder communication effectiveness. Additionally, these five EQ dimensions are linked to how Corporate Visual Identity (CVI) supports reputation, using the theoretical framework by Van den Bosch et al. (2005), which asserts that well-managed CVI can enhance an institution’s visibility, distinctiveness, authenticity, transparency, and consistency.

## 2. Literature review

### 2.1 Higher education identity

The marketing and management literature has extensively explored the importance of the concept of corporate identity, referring to the features, characteristics, traits, or attributes of a company that are deemed essential, distinctive, and enduring ( Clark et al., 2020; Coman et al., 2021; He & Mukherjee, 2009). Corporate identity is an organization’s presentation to each stakeholder, making the organization unique and combining aspects of communication, design, culture, behavior, structure, industry, and organizational strategy ( Wu and Cheong, 2024). As marketing consensus emphasizes the inside-out approach ( Clark et al., 2020), it is crucial for HEIs to understand the process of identity formation at the organizational level. Key stages in Visual Identity (VI) aim to integrate the institution’s values and vision into public and internal perceptions ( Iglesias et al., 2020; Siyanbola & Adeyemi, 2021). Furthermore, organizational identity is shaped through a combination of internal branding and VI ( Siyanbola & Adeyemi, 2021). Internal branding refers to an organization’s efforts to strengthen employees’ emotional attachment and understanding of its values ( Balmer and Podnar, 2021; Barros-Arrieta and García-Cali, 2021). This critical process ensures that all HEI members, particularly educators and staff, comprehend and embody the institution’s core values in every interaction with students and other stakeholders ( Clark et al., 2020). In other words, internal branding is the foundational basis of an HEI’s identity, which is then expressed through VI. Meanwhile, VI, encompassing elements such as logos, colors, and graphic symbols, plays a crucial role in conveying an authentic and consistent visual message of the HEI’s identity to both external and internal audiences ( Wu and Cheong, 2024). Various studies have highlighted that this visual consistency is necessary to build trust and a strong reputation in the eyes of the public ( Gregersen & Johansen, 2022). As an integrated element in organizational communication, VI becomes a tangible manifestation of the institution’s values and essence, with direct implications for the reputation of HEIs ( Del-Castillo-Feito et al., 2019).

However, previous studies on the formation of HEI identity have often overly emphasized aspects of brand image and reputation (e.g., Del-Castillo-Feito et al., 2019; Lafuente-Ruiz-de-Sabando et al., 2018; Miotto et al., 2020). An excessive focus on external perceptions can lead to the neglect of Visual Identity (VI) at the organizational level, as also highlighted by Gregersen and Johansen (2022). This poses a risk of weakening HEI branding overall, as VI is not merely a visual display but a reflection of the organization’s commitment to its values. Clark et al. (2020) show that VI is often considered secondary to other branding aspects, even though VI has a long-term impact on how HEIs are identified and recognized by the public. The oversight of VI in various studies indicates a deeper gap in understanding its role at the organizational level. Meanwhile, VI is not simply an external promotional tool but also an element that aligns internal identity with external perceptions ( Ali et al., 2022). A representative VI fosters strong internal organizational attachment, increases loyalty, and builds internal cohesion, which has a significant impact on the overall reputation of HEIs ( Ali et al., 2022; Mohamad et al., 2017).

A more comprehensive theoretical integration is necessary to deepen the understanding of VI within the context of HEIs. While existing research has primarily linked VI to reputation and external perception ( Clark et al., 2020; Gregersen & Johansen, 2022), a more substantive theoretical framework should explore its relationship with specific academic outcomes and recruitment metrics. For instance, strong VI can enhance prospective student attraction and enrollment rates, reinforcing institutional competitiveness ( Hemsley-Brown & Oplatka, 2022). Additionally, VI contributes to faculty and staff retention by fostering a sense of belonging and institutional pride, which, in turn, influences academic performance and research productivity ( Clark et al., 2020). The alignment between VI and institutional values not only strengthens internal identity but also supports broader strategic goals, such as student engagement and alumni involvement ( Ali et al., 2022). By integrating VI within a more extensive theoretical framework that connects branding with measurable HEI performance indicators, future research can bridge the existing knowledge gap and offer a more holistic perspective on its organizational impact ( Miotto et al., 2020; Mohamad et al., 2017).

In the modernization of higher education, institutional identity is shaped through four main structures: communication, behavior, organizational culture, and market conditions ( Melewar & Jenkins, 2002; Troyan & Kravchenko, 2021). These structures work together to build the institution’s image and reputation, involving the perceptions of both internal and external stakeholders ( Alessandri et al., 2006; Ribeiro et al., 2022). Visual Identity (VI), consisting of elements such as names, logos, color schemes, typography, and slogans, is a strategic asset that reflects the core values and character of the organization, while also strengthening recall and distinguishing the institution in the market ( Van Den Bosch et al., 2005; Foroudi et al., 2017). The logo, as the primary expression of visual identity, is not only a differentiating tool but also a communication medium that represents the organization’s ideas and qualities ( Van Riel & Van den Ban, 2001; Melewar & Akel, 2005). Institutional management uses the logo to support strategies, achieve goals, and articulate the competitive characteristics of the institution. In this context, the consistent management of VI can produce a cohesive and authentic identity narrative, enhancing the reputation and competitiveness of the institution in the increasingly competitive higher education sector ( Gregersen & Johansen, 2022). Therefore, the integration of VI into the internal branding of HEIs is not merely cosmetic but a strategic element that strengthens identity and relationships with stakeholders, creating sustainability and visibility that is widely recognized.

### 2.2 The important role of employee in internal branding

Dean et al. (2016) emphasize that employees play a crucial role in creating brand meaning through their experiences and interactions, making internal brand imaging essential for Higher Education Institutions (HEIs). Effective brand identity management in this sector requires in-depth marketing strategies, an understanding of the internal market, and the development of shared brand meaning to remain globally competitive. Involving employees in this process is critical because they act as brand representatives, and their motivation is crucial in conveying a consistent brand message ( Van Den Bosch et al., 2005). Additionally, the complexity of HEIs further underscores the importance of the internal market in realizing brand identity, although this often presents a challenge in developing a cohesive brand meaning ( Dean et al., 2016). Moreover, the complexity of the higher education market, with its perceived high risks and long-term consequences for consumers, hinders the effectiveness of traditional brand imaging approaches ( Mampaey et al., 2020; Subbarayalu, 2022). Branding in higher education is often impeded by internal barriers, such as the presence of multiple sub-brands, complex brand architecture, or the need for efficiency in university mergers, particularly in multi-campus universities ( Mampaey et al., 2020; Wu & Cheong, 2024).

Meanwhile, Clark et al. (2020) explore the concept of internal branding, which is closely related to but distinct from employer branding. Internal branding goes beyond building a brand through attractive logos and slogans ( Barros-Arrieta & García-Cali, 2021); it involves designing elements that embody the values upheld by the institution, ensuring that employees communicate the brand promise to customers through service delivery ( Foster et al., 2010). Therefore, employees have a direct influence on brand perception and enthusiastically identify with the institution’s identity, enhancing branding activities while acting as a crucial bridge between the institution and customers ( Bolhuis et al., 2018; Subbarayalu, 2022; Wu & Cheong, 2024). Additionally, HEI employees, as tangible assets, play a vital role in brand development and must be actively involved in shaping and promoting the brand ( Miotto et al., 2020; Slade-Brooking, 2016). This requires an integrative framework that connects human resource management and marketing, particularly in internal marketing communication to influence the brand promise ( Clark et al., 2020). In higher education, this is considered more challenging due to internal resistance ( Gregersen & Johansen, 2022). As Clark et al. (2020) indicate, the presence of multiple sub-brands and complex brand architecture often hinders effective internal branding, a challenge frequently encountered in HEIs.

In this vein, an effective internal branding strategy is crucial for fostering aligned brand expectations ( Dean et al., 2016). Furthermore, Dean et al. (2016) identified four stages in the brand cycle—awareness, interpretation, appropriation, and communication. In this brand cycle, employees must engage in each stage of the brand creation process, where internalization includes both macro and micro cycles of brand meaning. The macro cycle is shaped by employees’ historical brand experiences and the internal brand imaging efforts they encounter. In contrast, the micro cycle involves a personal dialectical process, where individuals assess and reinterpret brand information based on their interactions. As a result, employees’ understanding of the brand emerges through the combination of these macro and micro cycles, which illustrate the impact of internal brand imaging on how employees perceive and interpret the brand. Consequently, their historical brand knowledge (macro cycle) is continuously updated through ongoing evaluations and their personal experiences (micro cycle) with the brand. Therefore, in this study, the “experiential brand meaning” cycle is integrated with the Expressiveness Quotient (EQ) or five dimensions of reputation to categorize questions concerning the relationship between visual identity and employee involvement in its management.

### 2.3 Expressiveness Quotient (EQ)

The literature on HEIs’ reputation has revealed several determining factors used by stakeholders to assess reputation ( Dumitriu, 2018; Dursun and Altin Gumussoy, 2021; Foroudi et al., 2019; Panda et al., 2019). Meanwhile, previous research has rapidly developed regarding how HEI reputation is assessed based on measurable dimensions. For instance, Dumitriu (2018) employed the Quality Function Deployment method to identify key issues that require immediate improvement or change in terms of educational service quality. Additionally, Foroudi et al. (2019) highlighted the role of students in value co-creation concerning the significance of university website features. Dursun and Altin Gumussoy (2021) identified important factors influencing HEI reputation in the perceptions of students, alumni, and academic and administrative staff. Panda et al. (2019) conceptualized university brand image as heritage, service quality, and trust, and explored its relationship with student satisfaction. Furthermore, Amado-Mateus et al. (2024) evaluated the psychometric properties of a reputation scale among students at private universities in Colombia. However, these studies primarily focus on perceptions specific to HEI reputation, neglecting HEI identity as a reinforcement of reputation at the organizational level. To build a strong reputation, businesses must develop emotional appeal and express themselves to stakeholders in a credible, authentic, sincere, and convincing manner, clearly communicating who they are, what they do, and what they stand for ( Fombrun and Van Riel, 2004; Mohamad et al., 2017).

Therefore, in building a strong reputation, the involvement of HEI identity must be incorporated, impacting the flow of brand meaning formation at the organizational level. As emphasized by Van den Bosch et al. (2005), VI can enhance interconnected dimensions. In this vein, this section outlines the Expressiveness Quotient (EQ) proposed by Fombrun and Van Riel (2004), which is used to formulate questions related to strategic visual identity. The higher the expressiveness, the more likely it is to appeal to stakeholders ( Fombrun & Van Riel, 2004). This approach allows organizations to cultivate emotional appeal and to effectively understand, strategize, and execute reputation management ( Angliss, 2022). The first dimension, visibility, measures the brand’s presence in customers’ minds. According to Fombrun and Van Riel’s (2004) model, this can be assessed through street exposure, national heritage, media exposure, brand equity, listing on a public stock exchange, and corporate citizenship. Distinctiveness represents the organization’s unique position in the minds of customers and other stakeholders. Strategic alignment, emotionally appealing features, and attention-grabbing messages can achieve distinctiveness, allowing an organization to stand out in a crowded market. Transparency is another critical dimension, where greater transparency leads stakeholders to rely more on an organization’s disclosures, which are measured by products and services, vision and leadership, financial performance, social responsibility, and the working environment.

Additionally, an HEI’s identity can contribute to transparency by clearly communicating its values and commitments. Fombrun and Van Riel (2004) address authenticity as a challenge through four lessons: clarifying who you are, developing broad consensus within the organization, expressing your identity clearly, and remaining true to that identity. Authentic organizations are perceived as genuine, accurate, dependable, and trustworthy. Visual elements or logos that trace back to a company’s origins can enhance perceived authenticity, even if authenticity is not always directly related to VI. Consistency, the final dimension, is determined by consistently using brands and graphic elements over time. This consistency ensures that the brand’s identity remains clear and recognizable to its audience. In this study, the EQ was employed to formulate questions related to strategic visual identity elements. The decision to use the EQ was based on its five interconnected dimensions. Assessing the effectiveness of the measures undertaken is essential to building a robust reputation, and the EQ provides a comprehensive evaluation framework for this purpose. Van Den Bosch et al. (2005) explored the role of VI in reputation management, demonstrating how it aligns with corporate reputation through the interrelated dimensions in Fombrun and Van Riel’s (2004) reputation model. Managing VI effectively is crucial, as it represents an organization and serves as a visual expression intricately linked to its reputation.

## 3. Methodology

### 3.1 Research design

This study employs a qualitative case study approach as an investigative strategy, designed to explore contemporary phenomena in-depth within their real-world contexts. The case study method is particularly suitable when the boundaries between the phenomenon and its context are not clearly defined, enabling a comprehensive examination of intricate relationships and dynamics. Drawing on the structured guidelines of Creswell and Creswell (2018), this approach integrates multiple data collection techniques, including semi-structured interviews, direct observations, and document analysis, ensuring a holistic understanding of the research topic. The rationale for adopting the case study design stems from its ability to generate in-depth insights into the research context while accounting for its complexity. According to Yin (2018), the case study is especially valuable in investigating contemporary issues where traditional experimental controls are impractical. This research design further aligns with the recommendations of Elifneh (2017) and Yin (2018), as it emphasizes a holistic methodology to capture diverse perspectives and contextual subtleties.

The data collection process was conducted over a six-month period, during which semi-structured interviews were carried out with purposively selected participants representing a range of perspectives relevant to the research context. Interview questions were designed to be open-ended, enabling participants to provide rich, detailed narratives. Observations were documented systematically in a field journal, capturing contextual nuances and participant interactions. The document analysis focused on reviewing policy documents, institutional reports, and archival records, providing additional layers of context. To minimize bias and enhance the credibility of findings, this study employed methodological triangulation, which involves using multiple data collection methods to validate and cross-check results ( Creswell & Creswell, 2018). By integrating interviews, observations, and document analysis, the study ensured a more comprehensive and reliable understanding of how VI is managed and perceived within Indonesian HEIs. Data transcription was carried out using professional software to enhance accuracy, and member checking was conducted to validate the interpretations. Further, the results from these three methods were systematically compared and integrated through the following process:

a) Cross-validation: Responses from interviews were compared against observations and documents to identify consistencies and discrepancies. For example, if an interviewee claimed that their institution had a strong VI strategy, but observations revealed inconsistent branding elements across different units, this discrepancy was noted for further investigation.
b) Triangulated themes: Recurring themes across interviews, observations, and documents were identified. These themes included the role of VI in institutional reputation, internal branding efforts, and alignment between official branding policies and practical implementation.
c) Discrepancy resolution: In cases where contradictions emerged—such as an employee describing strong internal branding efforts, while document analysis suggested weak formal guidelines—follow-up discussions or secondary document reviews were conducted to clarify the inconsistencies.
d) Final synthesis: Data were synthesized into a coherent narrative, ensuring that each finding was supported by evidence from at least two independent sources.

This methodological triangulation strengthened the study’s validity by ensuring that conclusions were not solely reliant on a single data source, reducing potential researcher bias and increasing the robustness of the findings ( Creswell & Creswell, 2018).

### 3.2 Data collection instrument and participant

This study involves interviews with 29 employees from five public universities with legal entity status in Indonesia, known as PTNBH. Interviews were chosen as the most effective approach for collecting qualitative data through in-depth questions, which are essential for gathering qualitative information ( Van den Bosch et al., 2005). The selection of participants for this study followed a purposive sampling strategy, ensuring that the respondents had relevant experience and insight into the branding and VI management within their respective HEIs. Purposive sampling is commonly used in qualitative research to select individuals who can provide in-depth information relevant to the research problem ( Creswell & Creswell, 2018). The inclusion criteria focused on employees in non-academic positions who were actively involved in administrative, managerial, and strategic roles within the university. The participant group consisted of 13 male and 16 female members holding non-academic positions, such as heads of departments or unit managers at their respective public universities, which are legally established (see Table 1). Regarding respondent involvement, this study obtained verbal consent, with participants willingly agreeing to participate after being informed that the data provided would be used solely for academic research purposes, and their personal information would be kept confidential. The study also received ethical approval from the Directorate of Research and Community Service (DRPM) of Institut Teknologi Sepuluh Nopember, with approval number 2402/ITS.IX.8/B/PP.05.02.00/2022. Thus, all participants provided informed consent prior to their involvement in this study, and the research was conducted in accordance with the established ethical standards.

**Table 1.** Details of participants.

State University	Units	Occupation	Year at University	Alumni	Gender
1	Directorate of Student Affairs	Scholarship Manager	10	Y	M
	Directorate of Education	Director	37	Y	F
	Directorate of Community Service and Empowerment	Head of Sub Directorate	14	Y	F
	Sub-Directorate of Apprenticeships and Industrial Relations	Head of Sub Directorate	31	Y	F
2	Directorate of Student Affairs	Section Chief	30	N	M
	Directorate of Partnerships, Alumni and International Affairs	Section Chief	15	Y	F
	Directorate of Human Resources	Section Chief	23	Y	F
	Information Services Sub-Section	Head of subsection	18	Y	F
3	Directorate of Education	Head of secretariat	22	Y	M
	Directorate of Student Affairs	Head of secretariat	17	Y	M
	Institute for Research and Community Service	Head of Division	19	Y	M
	Directorate of Facilities and Infrastructure	Head of secretariat	33	N	F
	Institute for Innovation and Entrepreneurship Development	Head of Division	8	Y	M
	General Administration and Information Office	Head of Division	15	Y	F
	School of Business Management (SBM)	Marcomm Manager	13	Y	F
4	Directorate of Education	Section Chief	13	Y	M
	Directorate of Student Affairs	Head of Sub Directorate	30	Y	M
	Directorate of Human Resources	Section Chief	14	Y	M
	Directorate of Facilities and Infrastructure	Section Chief	8	Y	M
	*Airlangga Global Engagement* ( *AGE*)	Marcomm Manager	2	Y	F
	Directorate of Career Development, Entrepreneurship Incubation and Alumni (Dpkka)	Marcomm Manager	4	Y	F
5	Directorate of Student Affairs	Head of Division	18	Y	F
	Directorate of Business Cooperation and Management	Head of Division	26	N	M
	Directorate of Human Resources and Organisation	Head of subsection	17	Y	F
		Head of Division	19	Y	F
	Global Language Center Unit	Head of subsection	21	N	M
	Interdisciplinary School of Technology Management	Head of Division	30	N	M
	Innovation and Science Technology Area	Head of Division	29	N	F
		Head of subsection	14	N	F

The selection of participants aimed to ensure a representative sample from various units in each university. These employees were chosen because they are responsible for planning, strategy, and organizational policies. Diversity in selection was crucial, as it captured a range of experiences and perspectives regarding visual identity and organizational reputation. The study was conducted at five top-ranking PTNBH universities in Indonesia between 2021 and 2023, according to the Quacquarelli Symonds World University Rankings (QS WUR), Times Higher Education (THE), and Webometrics. Therefore, these universities were selected, including (1) Universitas Indonesia (UI), (2) Universitas Gadjah Mada (UGM), (3) Institut Teknologi Bandung (ITB), (4) Universitas Airlangga (Unair), and (5) Institut Teknologi Sepuluh Nopember (ITS).

Public universities with legal entity status in Indonesia were chosen as the subjects of this study because they have greater operational flexibility, particularly in academic and administrative areas, which are strictly regulated by their respective laws. Selecting the highest-ranked state universities with legal entity status not only simplifies the research process but also emphasizes the importance of global competition in higher education, where these institutions strive for excellence in international rankings. By focusing on these top-ranked public universities with legal entity status in Indonesia, the study examines institutions with significant societal influence, which are presumed to manage their visual identity (VI) meticulously. These rankings are crucial benchmarks for assessing quality and reputation, which are essential for stakeholders, including prospective students, governments, and institutions.

**Figure 1.**
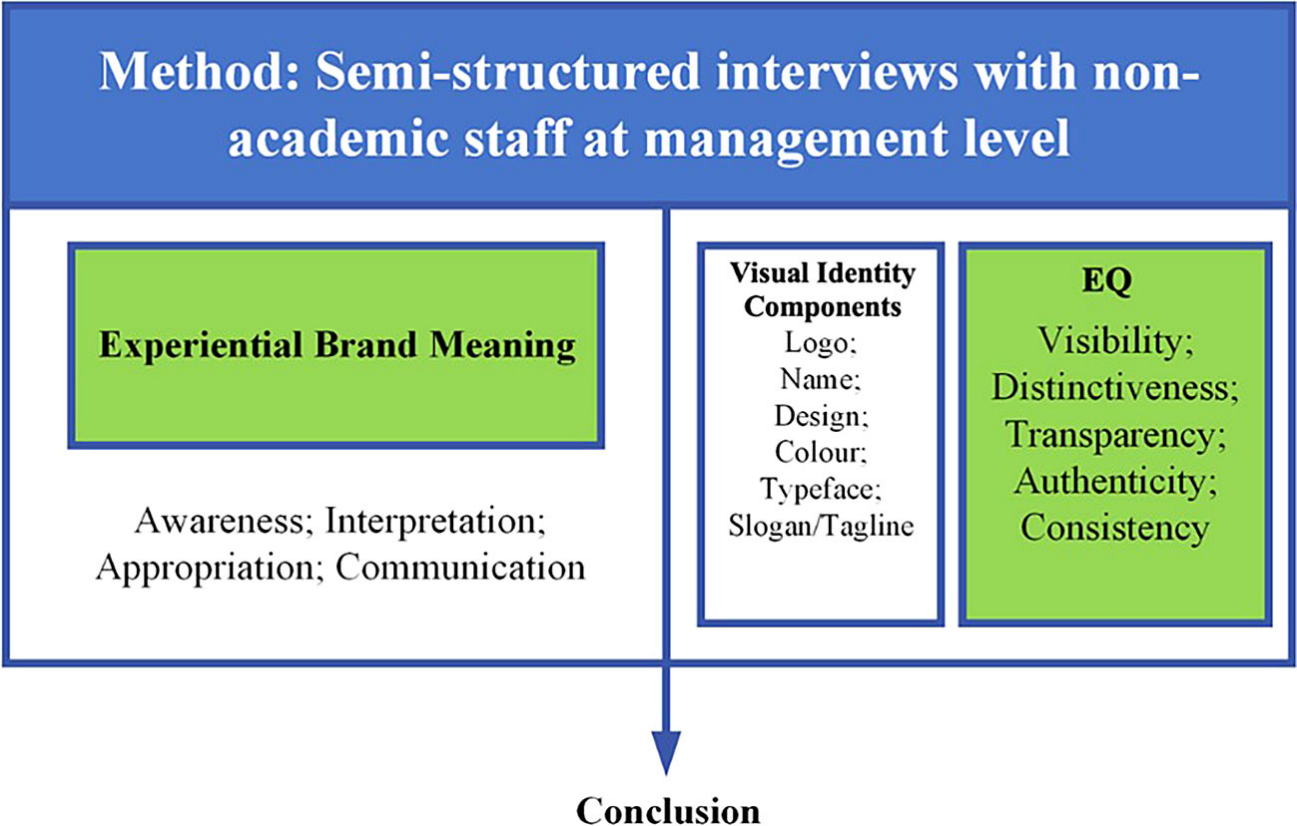
Semi-structured interviews.

Data collection for this study was conducted through semi-structured interviews (see Figure 1). These sessions were recorded and subsequently transcribed into written text for analysis. To ensure confidentiality, any personally identifiable information was carefully removed from the transcripts. The interview format was designed to explore the participants’ connection to the brand and their significant experiences. Each session lasted approximately 60 minutes and included semi-open questions focused on managing visual identity, derived from the five dimensions of reputation. This study focuses on the visual identities of organizations, which encompass elements such as logos, colors, designs, and other visual components that collectively shape the image and brand of state universities with legal entity status. Emphasizing visual identity underscores how these elements contribute to the overall perception and effectiveness of the university’s brand in the eyes of internal stakeholders. The approach highlights the critical role of visual identity in creating a cohesive and impactful brand within the university community and beyond (see Figure 1). It gathers diverse perspectives to deepen the analysis of institutional perceptions and the management of visual identity.

The interviews were structured around two sets of questions to explore different aspects of institutional branding and visual identity management. The first set focused on internal branding, specifically examining structural and organizational culture. Influenced by Dean et al. (2016), this part of the interview was based on the concept of “experiential brand meaning,” which outlines four stages in the brand cycle: awareness, interpretation, appropriation, and communication. This framework was used to explore how employees engage with each element in the brand-building process, shedding light on their commitment to embodying and promoting the brand’s values internally, within the context of universities in Spain. Similarly, Foroudi et al. (2020) and Punjaisri and Wilson (2011) explored how employees embody the brand. Clark et al. (2020) conducted previous research on the contribution of internal branding to university management in Canada. These studies emphasize the valuable role of internal branding in higher education brand management strategies. While there is substantial research on this topic in the context of universities in developed countries, there is a need for further research in developing countries such as Indonesia.

The second set of questions assessed employees’ awareness and understanding of institutional branding. These questions specifically targeted the interpretation, appropriateness, and communication of institutional identity, focusing on the logo and its components—name, design, color, typeface, and slogan, as outlined by Foroudi et al. (2020). This set was strategically designed to incorporate elements related to the five dimensions of reputation, facilitating a systematic exploration of the intricate relationship between visual identity and employee engagement. The interview questions were also tailored to uncover the multifaceted measures implemented to maintain a consistent visual identity, categorized under structural and cultural indicators. Organizational structure, as defined by Van den Bosch et al. (2004), refers to how an organization assigns its workforce to various tasks and coordinates its efforts effectively. According to Schein in the same source, culture is described as a set of fundamental assumptions shared within the organization. These assumptions evolve over time and help the organization address external adaptation and internal integration challenges. They are taught to new members as the correct way to perceive, think, and feel about these challenges. The development of organizational culture is significantly influenced by founders and leaders, whose practices in managing visual identity are deeply embedded in their ways of learning and influencing. This aspect of culture and leadership is crucial as it underpins the consistency of the visual identity that shapes public perceptions of the institution. In summary, the study examines how strategic branding efforts are integrated within the broader organizational framework.

### 3.3 Data analysis

The study utilized a qualitative inductive approach with data-driven thematic analysis, which involves detecting, analyzing, and reporting patterns or themes in the data ( Braun & Clarke, 2006). This method requires the researcher to sort through the data, looking for similarities or connections, a practice common to many qualitative research techniques ( Miles & Huberman, 1994). The findings are then presented in a narrative format, which is a typical method in case study research. This narrative approach helps to recreate the context and flow of evidence, allowing readers to better understand the meaning behind the data ( Czarniawska, 1997; Gillham, 2000). In addition, qualitative research methods, as noted by Strauss and Corbin (1990), are widely used because they enable the collection of in-depth data from fewer research units, rather than broad, surface-level information from many units ( Jennings, 2001). This type of analysis describes individual experiences, variations, and relationships, with a cross-case analysis offering multiple perspectives on a case, which helps answer the study’s research questions ( Creswell & Creswell, 2018; Yin, 2018). The cross-case comparison involved presenting the textual data consistently across all cases, which allows for a comprehensive view of the findings ( Gibbs, 2008).

## 4. Result and analysis

The aim of this study is to explain the correlation between visual identity components and reputation from the perspective of internal stakeholders. It concludes that the logo serves as an identity component that conveys the personality and values of higher education institutions to their employees. By unraveling the intricacies of the “experiential brand meaning” cycle—awareness, interpretation, appropriation, and communication—developed by Dean et al. (2016), this study examines the extent to which the identity of HEIs impacts experiential brand meaning for the educational staff of five state universities with legal entity status in relation to internal brand image. This, in turn, has implications for the understanding of visual identity within HEIs, which can create a brand image and reputation. Therefore, through the data analysis in this study, a number of interview results are categorized based on the themes of experiential brand meaning proposed by Dean et al. (2016).

### 4.1 Experiential brand meaning: Awareness

Based on interviews with various respondents, brand awareness prior to joining the university reveals that each new interaction with the brand begins with a micro-cycle of evaluation and interpretation. This study shows that exposure to brand elements, such as visual identity (VI), initiates the development of brand meaning. Some employees, even those who are not alumni of the university, were already familiar with its reputation and brand image without actively seeking information. Furthermore, after joining the university, employees strive to deepen their understanding of the HEI’s identity, emphasizing a sustained commitment to brand alignment and recognizing the importance of internalizing the institution’s values and identity.

*“I am quite familiar with this university, as information about it was often shared and embedded in my mind during my student years. In addition, this university is one of the best in Indonesia. Its reputation is widely known. The logo or visual identity certainly triggers brand meaning, as its philosophy is as strong as its reputation.”*

*“Although I already knew about the university, I strive to learn more deeply about the institution where I work, especially regarding the meaning of its visual identity, its application, university developments, rankings, and so forth, to internalize it better. This is important since I plan to serve at this institution for a long time.”*

Thus, this proactive approach to brand education reflects a solid internal culture that values and supports brand identity. It demonstrates that although VI elements are important, the overall reputation built across various channels plays a crucial role in brand perception. Furthermore, respondents view HEI identity as an organizational asset, recognizing its significant impact on overall reputation. This unanimous recognition reveals a well-embedded brand culture where the importance of a strong HEI identity is universally understood and valued. In this regard, respondents also noted that information on HEI identity can be accessed via the university’s website, which serves as a guide to its visual identity standards. Therefore, a high level of brand awareness and understanding is essential to ensure that HEI identity is conveyed consistently and effectively both internally and externally.

*"I believe that visual identity represents the brand’s identity, which can impact the university’s reputation. As employees, we need to understand its philosophy and application to maintain its reputation. The university provides a visual identity guide linked on the main website. If there is any confusion, we as employees can contact the management unit via email or WhatsApp."*

Upon recognizing the importance of new brand knowledge, individuals become motivated to move to the second stage, which involves interpreting the brand’s meaning.

### 4.2 Experiential brand meaning: Interpretation

At the interpretation stage, individuals engage in personal analysis and comprehension, representing a micro-cycle in the development of brand meaning. In this phase, they shift from passive recipients to active evaluators, critically assessing and interpreting the new brand knowledge they have acquired through interactions with the brand. Most interviewees offered positive endorsements for the brand, particularly within the context of their roles, indicating that the university brand is viewed as valuable and reputable in their work. This finding aligns with research on the dissemination of internal brand knowledge and its impact on employees. As noted by one respondent:

*"I always recommend the university’s brand, especially to alumni and partners, based on the knowledge I’ve gained during my time here, including information on rankings, research, and community service. It is part of my role and responsibilities to ensure the university’s reputation is positively regarded by alumni and partners."*

Sharing knowledge about brand meaning represents another significant area explored in this study. Most respondents actively shared their understanding of the HEIs brand with coworkers and colleagues within work-related units, a practice that reflects their awareness of the importance of disseminating brand knowledge and fostering a deeper understanding of brand identity within the organization, as emphasized by Mampaey et al. (2020). All respondents expressed that a clear and strong HEI identity is crucial to conveying the university’s distinctive characteristics.

*"Having a clear and strong identity is, of course, essential, as it is built on a philosophy and foundation established by the founders with great intention. Additionally, a strong and clear identity will naturally attract people’s interest, acting as an embodiment of the university’s characteristics."*

This unanimous perspective reflects a profound understanding that a strong brand identity is a valuable asset, crucial for communicating the university’s unique attributes and values. This shared belief underscores the importance of maintaining a consistent and coherent brand identity. Consequently, the interplay between stakeholders at both internal and external levels can lead to the co-creation of HEIs identity, which may not fully align with the core brand values. Rather, as this co-created meaning becomes accepted as the brand’s reality, the appropriation stage of the brand meaning reinterpretation cycle begins.

### 4.3 Experiential brand meaning: Appropriation

At the appropriation stage, employees form brand associations by internalizing the co-created brand meaning and developing brand engagement. Once they accept this co-created meaning as the brand’s reality, they fulfill their roles as brand interpreters ( Dean et al., 2016). Therefore, universities need to focus on brand reinforcement for their employees, as highlighted by some respondents who noted that HEIs have provided some form of brand education and training. However, other respondents indicated a lack of such provision. Effective brand reinforcement enhances employees’ understanding and behavior aligned with HEIs identity values.

*"Internalization, especially concerning campus branding, significantly impacts work scope and increases knowledge and pride in the university."*

The appropriation stage involves the internal and emotional processing of brand meaning, where employees develop brand associations that reflect their understanding of the brand’s purpose, personality, and values. This process fosters an emotional attachment to the brand, as the acquired meaning leads to a deeper relationship and engagement with it.

### 4.4 Experiential brand meaning: Communication

At the re-interpretation stage, communication is identified as essential for articulating and expressing brand meaning in each brand interaction. All respondents acknowledged the importance of HEIs identity in building relationships with stakeholders, particularly in promotional and collaborative activities. This universal acknowledgment highlights a comprehensive understanding of the critical role that brand identity plays in effectively engaging various stakeholders, as discussed in Foroudi et al. (2020).

*"The brand identity represented by a symbol or logo is crucial when building relationships with stakeholders, especially with government agencies. Our campus identity significantly influences collaborations as an educational institution. While this identity might be seen as bureaucratic, it also necessitates accountability to society, especially regarding partnerships. Therefore, I often convey this meaning when establishing partnerships, conducting research, or engaging in community service with partners, alumni, and others."*

*"I usually communicate the brand meaning during university events, visits, collaborations, community engagements, and other similar activities, as I am often asked about it."*

In this vein, employees actively communicate brand meaning to partners, demonstrating a proactive effort to convey HEIs identity. Consistency in brand messaging emerges as a significant theme among stakeholders during collaborations, visits, or specific events. By fostering a culture of consistent communication, the university can strengthen its brand identity and ensure that all stakeholders receive a coherent message. However, it is also necessary to consult with the visual identity (VI) manager to ensure the proper direction of VI usage. Some respondents frequently consult the VI manager to ensure precise implementation and communication.

*"I consult with the visual identity manager about publications and announcements. Since we often received feedback, we now regularly consult about its implementation to maintain consistency."*

This highlights the importance of consulting with the visual identity manager to ensure brand consistency. Thus, reinforcing HEIs identity at the organizational level is still needed to bridge this gap and ensure all employees accurately understand the values of VI. Consequently, this section illustrates that the effectiveness of the communication stage heavily relies on the thorough development of the preceding stages. While the communication stage completes the interpretation cycle, the evolving macro and micro brand meaning cycles continue. As one interpretation cycle ends, a new cycle begins, fueled by ongoing social interactions among diverse stakeholders. Employees articulate the brand through interactive dialogue, express their understanding of HEIs identity, and engage in re-evaluating the significance of VI knowledge. As a result, the experiential brand meaning cycle associated with VI and the brand persists, aligning with the framework proposed by Dean et al. (2016).

### 4.5 Expressiveness quotient: Visibility

Based on interviews with administrative staff at the structural level across five state universities with legal entity status, several insights emerged regarding brand visibility. For instance, University [1] faces challenges with its logo, which is often displayed in low-resolution media, diminishing its appeal and visibility. This issue highlights the need for high-quality media applications to ensure the logo is clear and impactful, as supported by Subbarayalu (2022), who discusses effective brand management and the importance of logo placement in educational institutions. In contrast, University [2] received positive feedback regarding brand exposure. Several employees noted that the university enjoys strong brand visibility across various media platforms, which significantly enhances the university’s public presence and recognition. Meanwhile, University [3] faces challenges with its slogan, which is perceived as less memorable due to its rare usage in brand communications and lack of integration with the logo. This gap affects impression and recall, underscoring the need for more consistent and strategic use of the slogan and logo to strengthen brand identity, as emphasized by Siyanbola and Adeyemi (2021).

Similarly, University [4] is known for its strong brand exposure across various media, positively contributing to its visibility. Consistent presence across these platforms helps maintain a strong brand image and demonstrates that the university’s media strategy effectively promotes its brand. However, University [5] faces various challenges, such as its name, logo, and slogan, which are still seen as hindering promotional and brand communication efforts. It is acknowledged that the visibility dimension reveals some challenges related to the brand visibility of HEIs, particularly regarding logos that often need to stand out more across different media. Addressing these challenges by enhancing logo application, ensuring high-quality media display, increasing brand exposure, making the slogan more memorable, and better integrating brand elements is essential for improving visibility and appeal.

### 4.6 Expressiveness quotient: Distinctiveness

Interviews with administrative staff at the structural level across five state universities with legal entity status revealed several findings related to the uniqueness of their brand elements. For instance, University [1] uses a typeface in its brand elements that is not perceived as evoking positive feelings, as it lacks a direct connection to the university’s brand. Therefore, a more suitable typeface that aligns with the brand’s character and carries stronger emotional appeal would be beneficial in enhancing the university’s uniqueness. Meanwhile, some employees at University [2] expressed dissatisfaction with their university’s logo, preferring logos of other universities they felt were more distinguished. This indicates an issue with the logo’s appeal and effectiveness as a brand differentiator. The university may need to evaluate the logo’s design to ensure it better represents the institution’s identity, is more acceptable to employees, and stands out among competitors.

Universities [3] and [5] face various challenges with their brand elements. Issues such as the logo being seen as unsuitable in a professional context, the university name lacking uniqueness due to regulations limiting names to geographical distinctions, and a name perceived as too long and complex detract from its simplicity. Additionally, brand colors are viewed as insufficiently unique and ineffective in influencing perception and behavior; the typeface is considered unremarkable, lacking appeal and connection to the brand; and the slogan is regarded as unmemorable, lacking emotional resonance, and insufficiently motivating within the work environment.

University [4] encounters challenges with brand colors that are perceived as less impactful on mood, as they are not popular among staff. This finding suggests that the university could consider brand colors with stronger emotional resonance to enhance overall appeal and uniqueness. The findings from these universities highlight the need for stronger, more culturally relevant brand elements that foster emotional engagement and alignment with the brand. Addressing these issues would allow universities to stand out in a competitive market and build closer relationships with stakeholders.

### 4.7 Expressiveness quotient: Transparency

In response to the increasing demand for transparency among Higher Education Institutions (HEIs) in a highly competitive environment, maintaining transparency has become a challenge impacting HEIs’ reputations. Based on this study’s analysis of interviews with educational staff at the structural level across five state universities with legal entity status, various perspectives on brand element transparency were revealed. At University [1], for example, there was a perceived lack of alignment between the brand colors and the HEI’s vision and mission. Some respondents expressed difficulty in associating the color scheme with the university’s core values, indicating a misalignment between the brand colors and the university’s identity. In contrast, Universities [2] and [4] demonstrated good levels of transparency, as the managerial level clearly communicated information related to products and services, vision and leadership, financial performance, social responsibility, and work environment. This comprehensive communication reflects a commendable level of transparency and indicates effective brand message management.

Meanwhile, Universities [3] and [5] face similar challenges related to their brand elements. For instance, the university names are perceived as misaligned with the intended message, particularly in emphasizing technology; the logo designs are seen as inadequate representations of higher education institutions; the colors are deemed inconsistent with the universities’ vision and mission; and the typeface and slogan are considered insufficient for conveying the desired message. Additionally, at University [5], some respondents reported limited awareness or clarity regarding brand implementation and a lack of oversight from related units in brand management, highlighting the need for improved management and supervision. These challenges underscore the need for a more cohesive brand design approach that aligns with the university’s messaging. Enhancing these elements would enable HEIs to more clearly convey their identity and values to stakeholders.

### 4.8 Expressiveness quotient: Authenticity

Authenticity shares a similarity with consistency, both emphasized as essential goals for HEIs. Gregersen and Johansen (2022) explicitly argue that a consistent Visual Identity (VI) must authentically reflect or express the essence of an HEI, essentially visualizing who or what the HEI represents. Meanwhile, in the current state of HEIs in Indonesia, this study reveals a range of internal organizational perspectives concerning the authenticity of university brand elements. University [2] stands out as the only institution receiving positive feedback regarding authenticity, as noted by respondents. The university is perceived to have a clear understanding of its roots and foundations, which are seen as authentic and trustworthy. Consequently, the HEI has successfully communicated its core values and origins, fostering a sense of reliability and authenticity. In contrast, respondents from University [1] observed that brand design elements were often inadequate in conveying a clear message and meaning. Frequently, the intended meaning requires further explanation to be fully understood, and the slogan is perceived as insufficiently specific in communicating the brand.

Respondents from Universities [3] and [5] highlighted similar issues to those noted previously, underscoring the need for a stronger, more coherent approach to managing brand elements to enhance authenticity. It is essential that these elements are clearly defined and communicated consistently to cultivate an authentic brand perception. Meanwhile, respondents from University [4] reported authenticity-related issues, such as a logo that stakeholders, both internal and external, found difficult to interpret, and a typeface that was seen as ineffective in communicating the university’s identity and was deemed inauthentic. Addressing these elements could therefore contribute to enhancing the university’s credibility and strengthening stakeholder trust.

### 4.9 Expressiveness quotient: Consistency

Consistency in Visual Identity (VI) is not solely about aesthetics; it is also closely linked to the perceptions and responses from both internal and external stakeholders. Inconsistencies in VI elements can lead to confusion and negative perceptions of HEIs, whereas alignment of VI elements enhances brand recall and recognition, creating a strong psychological bond. This study highlights several findings regarding consistency, as expressed by the respondents. Only University [2] has implemented clear and consistent VI guidelines, which support the reputation of the HEI. This underscores the importance of well-defined guidelines in maintaining a strong and cohesive brand image, ultimately enhancing credibility and trust among stakeholders.

In contrast, Universities [1], [3], [4], and [5] still require a more integrated branding approach to ensure that all elements are applied consistently. Addressing these inconsistencies will help build a more professional and reliable image. Furthermore, the findings of this study align with previous research on brand management ( Dwitasari et al., 2022) and maintaining a cohesive brand image ( Foroudi et al., 2020).

## 5. Discussion and Contribution

### 5.1 Discussion

This study makes a significant contribution to understanding the importance of visual identity (VI) in the formation of reputation and identity within higher education institutions (HEIs). The identity of HEIs plays a crucial role as the presentation of the organization to its stakeholders, encompassing communication, design, culture, behavior, structure, industry, and organizational strategies ( Wu & Cheong, 2024). Following the principle of marketing that begins from the inside out, it is vital for HEIs to build a comprehensive and integrated organizational identity through internal branding and VI. Internal branding aims to strengthen emotional attachment and employee understanding of the organization’s values, while VI, consisting of elements such as logos, colors, and graphic symbols, functions as an authentic and consistent visual communication tool ( Wu & Cheong, 2024). Therefore, this study employs the EQ framework to assess the experiential brand meaning at the internal level of HEIs in Indonesia.

The findings of this research indicate that awareness of identity and branding among internal stakeholders has a significant impact on shaping positive perceptions of the university’s brand identity, aligning with the findings of Buono and Fortezza (2017). Additionally, the study highlights positive reception toward HEI brand identity; however, differing perceptions among stakeholders necessitate further exploration to strengthen the brand identity and ensure alignment with stakeholder expectations. Before the brand is communicated to external stakeholders, it is essential for internal stakeholders to understand the brand as a reflection of the institution’s image. The findings also suggest that although there are initiatives for university branding training, understanding of core brand elements such as vision and mission is not yet evenly distributed among respondents. As Baker and Balmer (1997) assert, the evaluation of VI serves to identify organizational weaknesses. In this case, the logo, a key element of VI, serves as a valuable asset that creates brand recognition for the institution in an increasingly competitive market ( Van Den Bosch et al., 2005; Foroudi et al., 2020). Consistency in the application of VI is crucial for building and maintaining a strong reputation. The study finds a correlation between VI, particularly logos, and five dimensions of reputation: visibility, distinction, transparency, authenticity, and consistency. In terms of visibility, logo size on specific media presents a challenge that requires greater exposure. Meanwhile, the distinction aspect suggests that universities enhance their brand elements to create a more unique and positive impression, for example by improving logo design, color selection, more appealing typography, and a distinctive slogan.

Furthermore, the transparency aspect shows that some HEIs have effectively communicated information, but there is a need to ensure authenticity and effectiveness of brand elements in conveying the desired message. Regarding consistency, consistent efforts are necessary to ensure that brand elements are applied comprehensively across the institution and that academic staff understand the VI guidelines. Based on these findings, the study emphasizes that although VI contributes to building reputation, some universities need to address all dimensions of reputation more fully in order to be more effective in managing VI and strengthening the HEI brand in the eyes of stakeholders. Thus, the integration of VI into internal branding is not merely cosmetic but rather a strategic element that strengthens identity and relationships with stakeholders, creating sustainability and institutional visibility that is widely recognized. In short, visual identity is like a well-tailored suit—it’s not just about looking good; it’s about how well it fits the institution, and how it’s perceived by everyone from the internal team to the wider public. And if the fit’s off, well, we might need to swap a few elements around to make sure the message is clear, consistent, and confidently communicated.

### 5.2 Theoretical contribution

The theoretical contribution of the findings in this study to the field of higher education institution (HEI) identity, particularly in the context of visual identity (VI), can be seen through the development of understanding regarding the alignment between visual identity elements and experiential brand meaning. Based on the findings that reveal differences in the consistency and authenticity of VI elements among public universities, this study supports the view that VI is not only a symbolic identity but also a crucial instrument for effectively conveying the core values of the institution.

In the context of the Expressiveness Quotient (EQ) framework by Fombrun and Van Riel (2004), this study enhances the understanding of experiential brand meaning by emphasizing the importance of consistency and alignment of VI elements in creating a positive brand experience for stakeholders. The EQ framework, which highlights the role of brand experience in shaping perceptions and consumer satisfaction, suggests that visual alignment can strengthen perceptions of institutional quality in the minds of educational consumers, such as students and academic staff ( Dean et al., 2016). This study also contributes to supporting prior research that underscores the importance of consistency in corporate visual identity (CVI) to avoid confusion and inconsistency that could damage the institution’s brand image ( Clark et al., 2020; Coman et al., 2021; Van den Bosch et al., 2005). These findings emphasize that visual consistency not only enhances brand recall but also increases brand trust and stakeholder confidence, which are key aspects of HEI identity developed through cohesive brand experiences that align with the core values of the university ( Balmer & Podnar, 2021; Gregersen & Johansen, 2022).

Furthermore, this study expands the EQ framework in the context of HEIs by demonstrating that authentic VI elements that align with the institution’s mission and vision have the potential to strengthen experiential brand meaning. By focusing on consistency and authenticity in VI, HEIs can create a brand identity that is not only recognized but also valued by stakeholders, as consistent VI facilitates a brand experience that reflects the institution’s commitment to the quality and values of higher education ( Dean et al., 2016; Foroudi et al., 2020).

### 5.3 Practical contribution

The practical contribution of this study provides concrete guidance for academic staff, managers, and stakeholders in higher education institutions (HEIs) to strengthen the institution’s image and brand identity through consistency and authenticity of visual identity (VI) elements. For academic staff, they are encouraged to incorporate institutional visual elements, such as logos and colors, into their daily academic activities, thereby reinforcing the professional image of the institution. The findings encourage academic staff to understand and consistently apply VI guidelines in various academic activities and public communications. For managers, it is expected that they will develop clear visual guidelines and provide training for staff to ensure the consistent application of visual identity across all communication platforms. Managers should also monitor the implementation of VI regularly to identify and rectify any inconsistencies that may arise in various marketing materials and institutional communications. Meanwhile, other stakeholders, including alumni and industry partners, can support the institution’s positive image by adhering to visual guidelines in official activities and external communications. This strong and consistent visual narrative will help establish the institution’s image as professional and trustworthy in the eyes of the public.

## 6. Conclusion

This study aims to explore the level of knowledge, understanding, and employee engagement in comprehending the brand identity of five public universities (PTN-BH) in Indonesia. Using the theory of HEIs identity and the EQ (Expressiveness Quotient) framework to review the brand experience formulated by Dean et al. (2016), the study reveals that there are differences in the implementation of visual identity across each institution, particularly in the consistency of logo usage, colors, typography, and other visual elements. The findings indicate that visual consistency is crucial in building a credible and professional image, while the authenticity of visual elements helps to strengthen the overall identity of the institution. The study also highlights that universities with clear visual guidelines and strong internal communication are better able to maintain visual identity consistency, which in turn reinforces positive perceptions among stakeholders. In the context of the EQ framework, it was found that alignment of visual elements can create a positive brand experience, strengthen emotional connections, and enhance stakeholder engagement with the institution. This study also addresses a research gap in the existing literature, which generally focuses on the image and reputation of higher education institutions without emphasizing the importance of VI at the organizational level. The results of this study emphasize that consistent VI not only strengthens the institution’s image but also plays a role in creating a more meaningful brand experience for users, which ultimately enhances trust and stakeholder loyalty.

This study has limitations as it only focuses on five public universities in Indonesia, selected based on national and international rankings. The results may not reflect the situation in other institutions with different backgrounds and rankings. Furthermore, this approach may overlook other variables that could influence the implementation of visual identity in HEIs. Therefore, further research is recommended to include a broader range of universities from various categories and regions, as well as to consider other contextual factors in the application of visual identity. Beyond its scope, this study also has several methodological limitations. First, the use of a qualitative case study approach, while allowing for an in-depth exploration of VI in HEIs, limits the generalizability of the findings. Since data were collected from a small number of universities, the results may not be directly applicable to institutions with different governance models, academic missions, or resource allocations. Future studies could incorporate a larger sample or mixed-method approaches, combining qualitative insights with quantitative data, to enhance the external validity of the findings. Lastly, this study captures VI implementation at a specific point in time, without considering how it evolves over the long term. Given the dynamic nature of university branding, a longitudinal perspective would provide deeper insights into how VI strategies develop, adapt to changing external factors, and impact institutional reputation over time. Future research should explore VI from a longitudinal perspective to assess its long-term effectiveness in shaping university identity and competitiveness. By addressing these limitations, future studies can contribute to a more robust and nuanced understanding of VI in HEIs, strengthening both theoretical and practical knowledge in higher education branding.

## Ethical approval

This study received ethical approval from the Directorate of Research and Community Service (DRPM) of Institut Teknologi Sepuluh Nopember, with approval number 2402/ITS.IX.8/B/PP.05.02.00/2022. The research adhered strictly to the principles outlined in the Declaration of Helsinki, ensuring the highest ethical standards in research involving human participants. All participants were informed about the purpose, procedures, and potential risks of the study. Written informed consent was obtained from all participants prior to their involvement, ensuring their voluntary participation. Participants were assured of confidentiality and anonymity, and they retained the right to withdraw from the study at any time without any repercussions. The collected data were securely stored and used solely for the purposes of this research.

## Author contributions

Conceptualization: Putri Dwitasari, Ellya Zulaikha

Methodology: Putri Dwitasari

Data curation: Putri Dwitasari, Syarifa Hanoum, Rabendra Yudistira

Alamin

Investigation and Anlysis: Putri Dwitasari, Ellya Zulaikha, Syarifa Hanoum

Preparation of the manuscript: Putri Dwitasari, Syarifa Hanoum, Rabendra Yudistira

Alamin, Luqman Lee

Revision of important intellectual content: Putri Dwitasari, Luqman Lee

Supervision: Ellya Zulaikha

## Data Availability

This study ensures the security and confidentiality of the data, as it involves sensitive organizational information that is classified and proprietary to the organization being studied. Since the interview participants include members of the organization’s management, there is a potential risk of reputational impact, and disclosure of the information could violate ethical research principles. To address this, the researchers have assured all participants that the data will be securely protected and used solely for research purposes. For those wishing to access the data, a formal request must be submitted to the corresponding author, accompanied by evidence of ethical approval and a detailed data access application. The data supporting the findings of this study can be accessed by contacting the corresponding author ( putridwita@its.ac.id). Requests for data access will be considered if intended for non-commercial research purposes, with the condition that proper credit is given to the original authors.
