# Supplementary material for: Internal Perspectives on Visual Identities in Higher Education: A Case Study of Top-Ranked Universities in Indonesia
Source: F1000Res. 2025 Mar 26;13:1535. Originally published 2024 Dec 19. [Version 2] doi: 10.12688/f1000research.159232.2 (PMC11729185; doi:10.12688/f1000research.159232.2)
Supplement: Peer review history

# Reviewer report: Reviewer response for version 1

**DOI:** 10.5256/f1000research.174929.r356450

**Published:** 2025-02-07

**Authors:** Katelijn Quartier

**Affiliations:** 1 Hasselt University, , Hasselt, Belgium

The article describes a study on the importance and impact of visual identity (VI) on higher education. What makes this study special is that it looks not at how it is perceived by externals but how it is perceived internally. Two previously published dimensions are used for this purpose: the Expressiveness quotient and the experiential brand meaning. The study concludes that VI is crucial to building a good brand that is lived out by everyone.

 The paper is very clear and well written with a good structure. The qualitative methodology is well justified and grounded in literature. Especially the discussion of the results is brought very enlightening. The quotes help to get a better grip on what the underlying idea is. The authors also acknowledge the limitations of their study which opens the door for further study.

 Some minor comments:

 Avoid repetition: for example at '4. Results' the whole first paragraph is already a summary of the previous text. This seems redundant to me.

 When you start explaining the dimensions of the Expressiveness quotient, list all five first so the reader is with you.

 In 4.6 the distinctiveness of the VI is discussed. I kind of missed seeing all five of them since they are discussed in detail: typography, color, ... It is hard to understand without seeing them.

Is the work clearly and accurately presented and does it cite the current literature?

Yes

If applicable, is the statistical analysis and its interpretation appropriate?

Yes

Are all the source data underlying the results available to ensure full reproducibility?

No source data required

Is the study design appropriate and is the work technically sound?

Yes

Are the conclusions drawn adequately supported by the results?

Yes

Are sufficient details of methods and analysis provided to allow replication by others?

Yes

Reviewer Expertise:

Retail and brand design

I confirm that I have read this submission and believe that I have an appropriate level of expertise to confirm that it is of an acceptable scientific standard.

# Reviewer report: Reviewer response for version 1

**DOI:** 10.5256/f1000research.174929.r354623

**Published:** 2025-01-13

**Authors:** Bing Bedjo Tanudjaja

**Affiliations:** 1 Petra Christian University, Surabaya, Indonesia

The journal can be accepted with minor revisions.

 In the data collection method there are several aspects that need to be considered such as Participant Selection please also explain how the participant selection process was carried out. For example, are there any specific criteria used to select these participants? then also related to Methodological Triangulation, explain further about how methodological triangulation was carried out in this study to add depth to the methodology. For example, how were the results from different methods (interviews, observations, document analysis) confronted and integrated?

 Also include Methodological Limitations: Discussion of the limitations of the research methodology used can be more explicit. For example, limitations in generalization from case studies, or potential bias in case selection. In the data collection method there are several aspects that need to be considered such as Participant Selection please also explain how the participant selection process was carried out. For example, are there any specific criteria used to select these participants?

 then also related to Methodological Triangulation, explain further about how methodological triangulation was carried out in this study to add depth to the methodology. For example, how were the results from different methods (interviews, observations, document analysis) confronted and integrated?

 Also include Methodological Limitations: Discussion of the limitations of the research methodology used can be more explicit. For example, limitations in generalization from case studies, or potential bias in case selection.

Is the work clearly and accurately presented and does it cite the current literature?

Yes

If applicable, is the statistical analysis and its interpretation appropriate?

Yes

Are all the source data underlying the results available to ensure full reproducibility?

Yes

Is the study design appropriate and is the work technically sound?

Yes

Are the conclusions drawn adequately supported by the results?

Yes

Are sufficient details of methods and analysis provided to allow replication by others?

Yes

Reviewer Expertise:

Visual Identities, Visual Culture, Visual Communication Design

I confirm that I have read this submission and believe that I have an appropriate level of expertise to confirm that it is of an acceptable scientific standard.

# Reviewer report: Reviewer response for version 1

**DOI:** 10.5256/f1000research.174929.r354625

**Published:** 2025-01-10

**Authors:** Teofilus Teofilus

**Affiliations:** 1 Ciputra University, Surabaya, Indonesia

The article "Internal Perspectives on Visual Identities in Higher Education: A Case Study of Top-Ranked Universities in Indonesia" presents a comprehensive examination of the influence of visual identities (VI) on institutional reputation within higher education institutions (HEIs). Through semi-structured interviews with employees from five leading Indonesian universities, this study evaluates the role of VI through the Expressiveness Quotient (EQ), emphasizing dimensions such as visibility, distinctiveness, transparency, authenticity, and consistency. The findings suggest that effective management of VI aligns internal stakeholder perceptions with external branding, thereby enhancing competitive advantages and fostering cohesive organizational identities.

**Constructive criticisms and recommendations**

1. Depth of Theoretical Integration: This article would benefit from incorporating a more comprehensive theoretical framework by establishing connections between VI's impact and not only reputation but also specific academic outcomes or recruitment metrics, thereby providing a more substantive link to HEI performance.
2. Broader Empirical Evidence: While the case study approach provides in-depth insights, incorporating quantitative elements can enhance the generalizability of the findings. Subsequent research might consider employing surveys or experimental designs to validate qualitative insights on a larger scale.
3. Comparison with Global Standards: This study focuses on Indonesian universities but lacks a comparison with global practices. Incorporating examples or data from Higher Education Institutions (HEIs) in other regions could enhance the analysis and provide a benchmark for Indonesian universities.
4. Stakeholder Diversity: Expanding the range of interviewees to encompass external stakeholders, including students, alumni, and industry partners, could provide a more comprehensive perspective on how VI influences perceptions beyond the internal staff.

To ensure the scientific validity of this article, addressing these issues is essential. Expanding the theoretical framework, implementing a mixed-methods approach, diversifying the participant base, and incorporating comparative global insights would substantially enhance the methodological rigor and generalizability of the research.

Is the work clearly and accurately presented and does it cite the current literature?

Yes

If applicable, is the statistical analysis and its interpretation appropriate?

Yes

Are all the source data underlying the results available to ensure full reproducibility?

Yes

Is the study design appropriate and is the work technically sound?

Yes

Are the conclusions drawn adequately supported by the results?

Yes

Are sufficient details of methods and analysis provided to allow replication by others?

Yes

Reviewer Expertise:

Higher Education, Strategic Management, Consumer Behavior

I confirm that I have read this submission and believe that I have an appropriate level of expertise to confirm that it is of an acceptable scientific standard.

## References

[1] : A review of higher education image and reputation literature: Knowledge gaps and a research agenda. *European Research on Management and Business Economics* .2018;24(1) : 10.1016/j.iedeen.2017.06.005 8-16 10.1016/j.iedeen.2017.06.005

[2] : Enhancing university brand image and reputation through customer value co-creation behaviour. *Technological Forecasting and Social Change* .2019;138: 10.1016/j.techfore.2018.09.006 218-227 10.1016/j.techfore.2018.09.006
